# A Bibliometric Study on the Evolution of Women’s Football and Determinants Behind Its Growth over the Last 30 Years

**DOI:** 10.3390/sports12120333

**Published:** 2024-12-03

**Authors:** Javier Ventaja-Cruz, Jesús M. Cuevas Rincón, Virginia Tejada-Medina, Ricardo Martín-Moya

**Affiliations:** 1 Department of Physical Education and Sports, Faculty of Education and Sport Sciences, University of Granada, Melilla Campus, ES-52071 Melilla, Spain; jventaja@ugr.es (J.V.-C.); rmartinm@ugr.es (R.M.-M.); 2 Melilla Football Federation, ES-52001 Melilla, Spain; 3 Department of Research and Diagnostic Methods in Education, Faculty of Education and Sport Sciences, University of Granada, Melilla Campus, ES-52071 Melilla, Spain; jcuevas@ugr.es

**Keywords:** female soccer, women’s sports, motivation, scientific production, Biblioshiny, science mapping

## Abstract

Background: The evolution of women’s football over the past three decades has been remarkable in terms of development, visibility, and acceptance, transforming into a discipline with growing popularity and professionalization. Significant advancements in gender equality and global visibility have occurred, and the combination of emerging talent, increasing commercial interest, and institutional support will continue to drive the growth and consolidation of women’s football worldwide. Methods: The purpose of this study was to present a bibliometric analysis of articles on the evolution of women’s football in terms of scientific production as well as its causes and motivations over the past 30 years (1992–2024). A total of 128 documents indexed in the Web of Science database were reviewed. Outcome measures were analyzed using RStudio version 4.3.1 (Viena, Austria) software and the Bibliometrix data package to evaluate productivity indicators including the number of articles published per year, most productive authors, institutions, countries, and journals as well as identify the most cited articles and common topics. Results: Scientific production on women’s football has shown sustained growth, particularly since 2010. Key research areas have focused on injury prevention, physical performance, psychosocial factors, motivation, and leadership. The United States, the United Kingdom, and Spain have emerged as the most productive countries in this field, with strong international collaboration reflected in co-authorship networks. Conclusions: The study revealed a clear correlation between the evolution of women’s football and the increase in scientific production, providing a strong foundation for future research on emerging topics such as the importance of psychological factors, sport motivation and emotional well-being on performance, gender differences at the physiological and biomechanical levels, or misogyny in social networks, thus promoting comprehensive development in this sport modality.

## 1. Introduction

In the past three decades, women’s football has experienced unprecedented growth both in terms of participation, with a 32% increase between 2010 and 2015, reaching 30 million participants, a figure that, according to FIFA estimates, will double by 2026 [1], and is closely linked to factors such as increased media visibility, the hosting of high-profile sporting events, the creation of professional leagues, and institutional support [2,3]. Moreover, global visibility has grown significantly in recent years, driven by FIFA and UEFA’s interest in increasing exposure for women’s football to unlock its commercial potential, resulting in a notable expansion of the fan base, with greater public interest and increased match audiences, both in attendance and TV broadcasts [4,5]. This phenomenon has been promoted by a combination of social, cultural, and institutional factors that have allowed this variant of football to move from a marginalized position to a central role in the international sports landscape, influencing the participation, performance, and overall experience of female players [6]. In this regard, other sports like rugby or women’s basketball have also experienced significant growth in the pace of professionalization, with the structure and support of the WNBA [7,8] or World Rugby, thanks to increased media coverage and global recognition. However, rugby still faces significant challenges in reaching the levels of women’s basketball or soccer [9]. Global competitions, such as the FIFA Women’s World Cup and the Olympic Games, have significantly contributed to its growth, attracting millions of viewers and increasing investment in the development of national and international leagues [10]. However, alongside the rise in women’s football practice and following, there has also been growing academic interest in studying this sport from a scientific perspective.

The evolution of women’s football and the increasing professionalization of sport have created a favorable environment for research in various fields [11]. In fact, both trajectories have grown similarly, from studies primarily addressing gender and inequality issues in sports [12,13] to those analyzing aspects related to physical performance [14,15], biomechanical aspects [16,17], physiological factors [18], psychological aspects [19,20], injury prevention [21,22], and sports management [23], among others. Therefore, the scientific production related to women’s football has grown considerably as it has gained visibility and professionalization, showing a drastic increase in football research in general since the year 2000, specifically by 94%, with 20% of that focused on women, particularly professional players [24], with descriptive studies predominating over experimental ones [25]. Additionally, a peak of publications has been observed in the last four years, representing 53% of the studies, indicating a clear upward trend and a growing interest from the scientific community [26]. The body of scientific literature reflects not only the rise of sport, but also an increasing concern with understanding the specific characteristics of women’s football and how it differs from men’s football in terms of performance, health, development, and management [27]. Similarly, an increased interest has been observed from researchers and sports clubs in the science of football as it relates to women. However, there is still inequality in the number of studies focused on female football players compared to their male counterparts, which highlights the need to continue promoting research in this area.

The motivations behind the growth of women’s football are multiple and diverse, ranging from the recognition of rights to changing cultural attitudes toward female athletes [28]. On the one hand, the support of sports and governmental institutions has been essential in providing the infrastructure, resources, and training for female athletes [11], although these commitments regarding funding are part of a broader strategy to accelerate the growth of women’s football and enhance the development and professionalization at a global level [10], inequality in this area still persists. On the other hand, it is crucial to analyze the individual experiences of the female players, coaches, and leaders who have played a key role in the expansion of women’s football [23]. The visibility of successful female role models has inspired younger generations to follow in their footsteps, benefiting from the exposure of high-profile athletes in their own sport [29].

Bibliometric studies are a valuable tool for analyzing the evolution of scientific production in a specific area, as they allow for the quantification and evaluation of publication trends, with quantity and quality indicators that include the number of publications, the impact of articles, or citations received; and structural indicators that measure the collaboration between authors and institutions and the evolution of research topics over time [30,31]. In the case of women’s football, a bibliometric analysis can identify the most researched fields of study, knowledge gaps, the most influential authors, and the leading institutions and countries in this sport’s research. Co-authorship network analysis can also provide insights into collaboration patterns among researchers, which is crucial for understanding how academic communities are forming around the study of women’s football [32]. In summary, such studies can offer a comprehensive view of how the growth of women’s football has been accompanied by increased scientific production and how advances in research have contributed to the sport’s development.

To date, two significant bibliometric studies have been conducted on this topic. The first one [24] examined the evolution of the scientific literature by comparing the focus on women’s football versus men’s football. It conducted a search for articles in the Web of Science database covering football in general and women’s football from 1970 to 2019, thus revealing gender disparities and the scarcity of studies on professional female players. The research highlighted that only 20% of the analyzed studies focused on women, and of these, only 15% focused on professional players. The remaining percentage exclusively represented men’s football, indicating the substantial disparity in gender representation within the scientific literature, largely due to the higher visibility and greater availability of resources for men’s football. The study underscores a lack of attention to areas such as match analysis and specialized training for professional female footballers, reflecting a clear underrepresentation of these topics in the existing literature, which significantly affects the amount of knowledge available. The second study [26] extended the analysis to 2024 using the Scopus database. This paper provided a performance analysis, thematic mapping, and a co-citation analysis to trace trends and future research areas. It stands out for its focus on international collaboration and the identification of emerging keywords in women’s football research, highlighting a significant increase in scientific production since 2019, with notable growth over the past four years, and identified the United States and the United Kingdom as the major contributors to this field of research. Both studies emphasized the need for greater academic attention to specific aspects of women’s football such as professional performance, injury prevention, and match analysis.

Therefore, the objective of this bibliometric study was to analyze the evolution of scientific production related to women’s football over the last three decades (1992–2024), providing a detailed overview of the research conducted to date and its impact on the sport’s development. Unlike previous studies [24,26], this study emphasized the capacity to establish a correspondence between the popularization and growth of the sport at the institutional, commercial, and cultural levels as well as the increase in scientific research. It aimed to accurately assess the productivity indicators by identifying the growth in the number of publications over time as well as the most influential journals, authors, and institutions in women’s football research. This included analyzing the most frequent research areas and exploring the networks of collaboration between researchers and geographic regions to understand how scientific communities have evolved around this topic. Additionally, the study offers an analysis of psychosocial factors, such as motivation and leadership, providing a more holistic perspective on the growth of women’s football. Although the studies address similar themes, they employ complementary approaches due to the use of different databases, thereby offering a distinct perspective in the analysis, making both valuable for understanding not only academic development, but also the factors driving the growth of women’s football.

## 2. Materials and Methods

### 2.1. Database and Search Strategy

In this study, a quantitative-bibliometric methodology was employed using the Web of Science (WoS) database. The search was conducted within its “Core Collection”, an index that ensures high quality in the results obtained. Several search terms were used, consistently including the terms “female soccer” and “motivation” as the primary search criteria in English, combined with the Boolean operators “OR” and “AND”, along with associated keywords (Table 1).

### 2.2. Selection of Articles for Bibliometric Analysis

For the bibliometric analysis, only original articles, review articles, early access articles, and book chapters were selected. Consequently, conference proceedings and publication retractions were excluded. No time limits were applied, allowing for the inclusion of all publications up to 31 July 2024, covering the period between 1992 and 2024. The final sample consisted of 127 articles.

### 2.3. Bibliometric Data Analysis

The bibliometric data analysis was conducted using the Biblioshiny application, which provides a web interface for Bibliometrix, an open-source tool used for quantitative research in bibliometrics, utilizing RStudio version 4.3.1 (2023-06-16 ucrt), Copyright © 2023, The Foundation for Statistical Computing Plattform, Viena, Austria [33].

Additionally, VOSviewer v.1.6.16 [34] was employed for constructing and visualizing graphs, network maps, and thematic maps [35]. Variables used in the analysis included sources of information, authors, countries, author keywords, and Keyword Plus (Table 2).

In this study, the overall performance of the research components was analyzed based on three criteria: sources, authors, and documents. The final analysis focused on the ten most relevant journals and authors, the author and country collaboration network, the ten most cited articles, the most productive countries and institutions as well as author keywords and Keyword Plus. The H-index of the leading journals and authors was also considered as an approximate measure of influence within the body of analyzed publications. The journal impact factor (JIF) was used as a measure of their scientific influence, with this information extracted from the metrics disclosed in the journal citation reports (JCRs) during the years in which the articles were published, using both the average JIF and the most recent one available, in 2023.

## 3. Results

### 3.1. Descriptive Analysis

Below is the key information about the sample scientific documents analyzed (Table 3). The collected data highlight the main sources of reference and the number of keywords in both versions (author keywords and Keyword Plus) as well as the number of authors and the primary variables investigated.

A total of 127 documents were published between 1992 and 2024, with 508 authors contributing over this period to 75 different sources. These publications included 335 author keywords. On average, the documents generated 36.54 citations per document during the evaluation period, with an average of 3.32 citations per year. There were only 7 individual authorships, indicating a limited number of solo publications, and the collaboration index was calculated to be 4.34, reflecting a high level of co-authorship. Additionally, 26.77% of the publications involved international co-authorship, indicating significant global collaboration in this research field.

### 3.2. Annual Scientific Production

**Figure 1:**
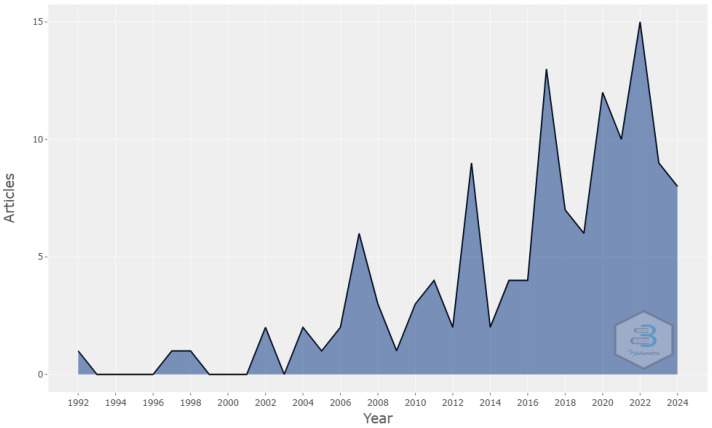
Annual scientific production (1992–2024).

Scientific research related to women’s football, motivation, or the reasons why women engage in the sport began to appear in the 1990s. An upward trend in the production of scientific articles was observed, with an annual growth rate of 7.11%. Figure 1 shows the evolution of the subject matter over time.

Until the year 2000, only three articles were recorded, and it was from 2002 onward that a progressive increase could be seen, reaching 2010 with a sixfold increase in production compared to the previous decade, with a total of 20 published documents. Although the growth was not linear, with fluctuations occurring every two years, 2007 stood out with an increase in production, reaching six manuscripts. Similarly, in the following decade (2010–2019), we observed nonlinear growth, with the most productive years being 2013 (n = 9) and 2017 (n = 12), accounting for a total of 52 articles over ten years. In the current decade (2020–2024), the trend continues with steady growth, with 2022 being the most productive year, recording 15 articles, and a total of 55 publications up to the date of analysis where women’s football is a central theme.

### 3.3. Most Relevant Journals

**Figure 2:**
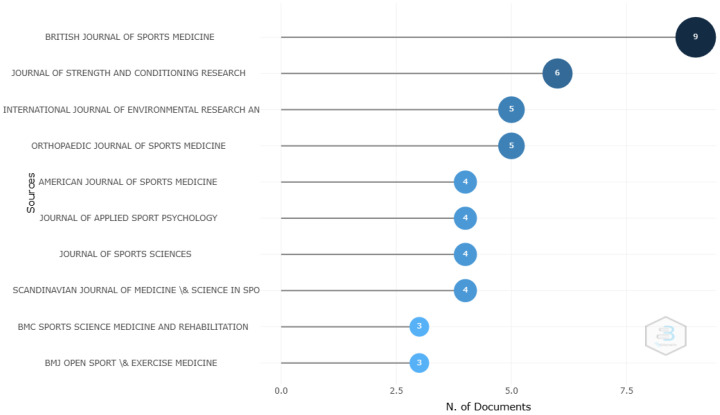
Top ten most relevant journals.

A total of 75 journals have published studies on women’s football, of which 60% are open access. This means that research on this topic is more easily accessible to a broader audience, which is particularly important in the field of sports, where findings should be shared not only with academics and researchers, but also with coaches, sports clubs, and even educational institutions. The importance of sharing this knowledge adds extra value to the contribution in advancing both sports and gender equality in a more inclusive and accessible way. Figure 2 shows the 10 most relevant sources in terms of scientific article production, all of which are open access. The most notable was the *British Journal of Sports Medicine*, with 9 published articles, an average impact factor of 7.67 since 2006, and the highest impact factor in 2023 (JIF = 11.8). It stood out with an H-index of 8 and a total of 949 citations. The descriptive information of the most important journals is presented in Table 4.

According to Bradford’s law, which describes the distribution of academic articles in scientific journals, articles on a specific topic are not evenly distributed among journals, but tend to concentrate in a few, known as “core journals”. It defines three zones: Zone 1, core journals; Zone 2, secondary journals; and Zone 3, peripheral journals [36].

**Figure 3:**
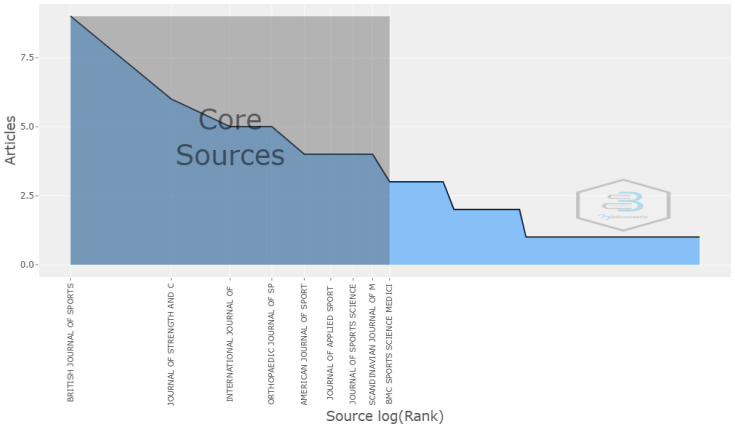
Relevant journals according to Bradford’s law.

**Figure 4:**
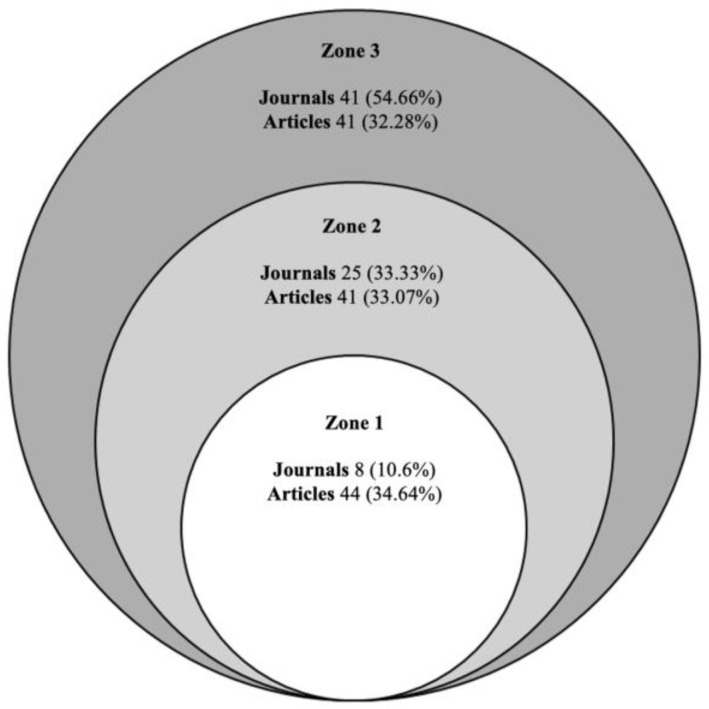
Distribution of scientific production according to Bradford’s law.

In the analysis conducted, 9 out of the 10 most relevant journals were present in the core (Zone 1), which were the most productive and concentrated most of the main articles (Figure 3). On the other hand, as observed in the graphical representation after applying Bradford’s law, the growth in the number of journals across the zones was exponential, while the distribution of articles was more balanced (Figure 4). In the core, 8 journals (10.6%) concentrated many of the articles (n = 44, 34.64%), whereas in Zone 3, the dispersion was greater, with 54.66% of the journals (n = 41) having only one publication each on the topic.

### 3.4. Most Relevant Authors

**Figure 5:**
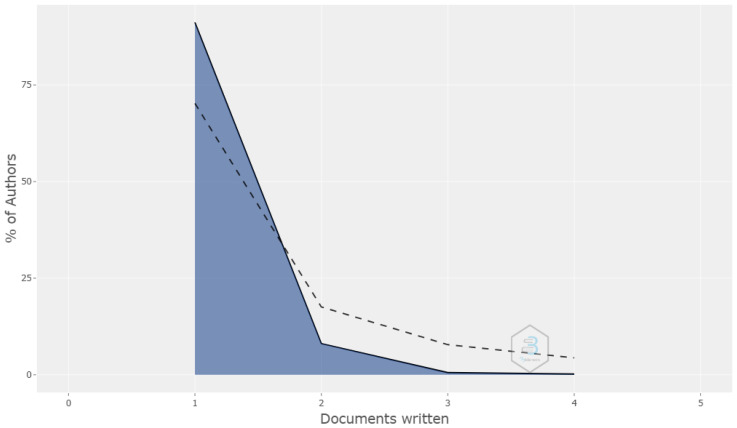
Author productivity according to Lotka’s law.

A total of 508 authors published 127 documents on women’s football, with an average of 4.16 authors per document. After applying Lotka’s law, which describes the distribution of scientific productivity among authors on a specific topic [37], it was estimated that 91.7% of these authors had made only one contribution to the field of women’s football, 7.7% made at least two contributions, and only 0.6% of the authors contributed three articles, as shown in Figure 5.

**Figure 6:**
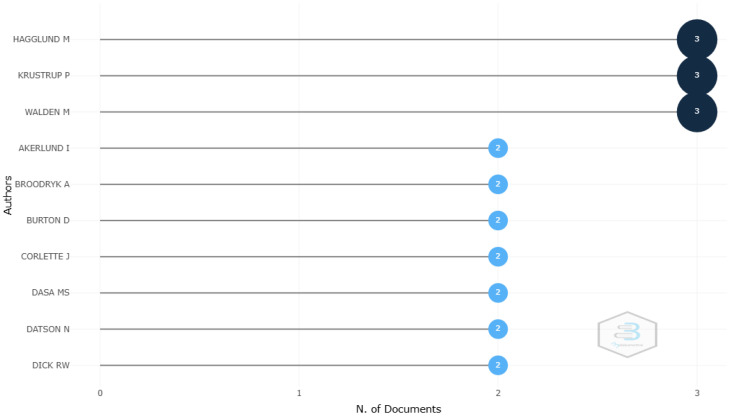
Top ten authors with the highest scientific production.

On the other hand, the ten most relevant authors in women’s football, with the highest number of published documents, are shown in Figure 6. The first group of authors with three publications included Hagglund, Walden, and Krustrup, while the rest had two documents each. The author with the most local impact was Krustrup, with an H-index of 3 and a total of 176 citations.

### 3.5. Production and Collaboration Between Countries

**Figure 7:**
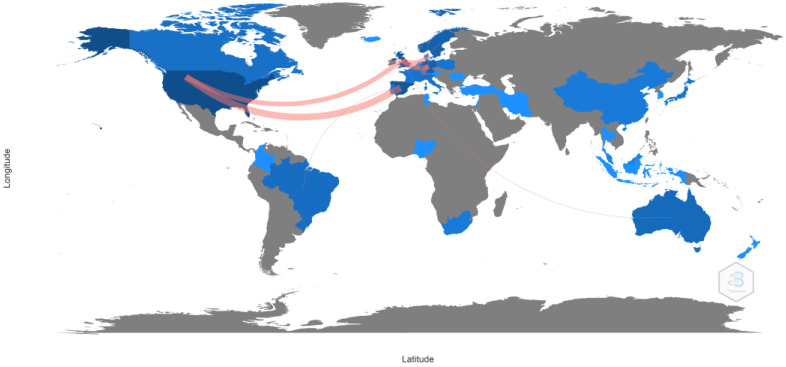
Collaboration map between countries.

**Figure 8:**
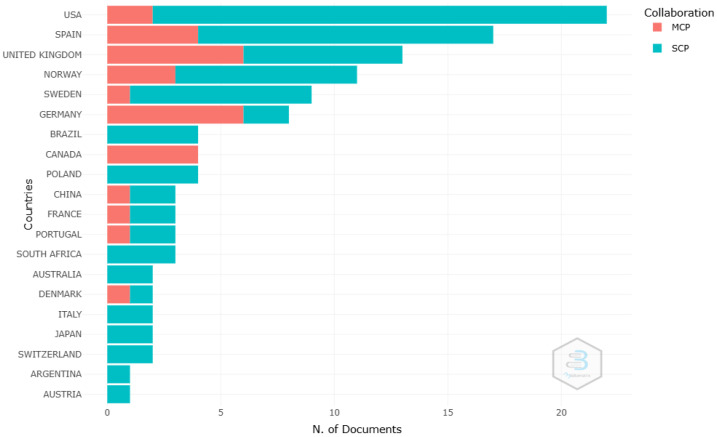
Country of the corresponding author. SCP: single-country publication; MCP: multiple-country publication.

Regarding scientific output by country, 33 countries contributed to publications in this area as well as to production resulting from key collaborations between the most relevant countries. The United States led the ranking with 106 publications, followed by the United Kingdom and Spain, with 58 and 55 documents, respectively. The three countries with the highest number of citations were the United States (n = 992), Spain (n = 645), and Sweden (n = 641) (Table 5). Additionally, the authorship collaboration map between countries as well as the country of the corresponding author reflected the involvement of many countries worldwide, highlighting the global nature of the topic studied and the interest it generates in the international scientific community. Notable data included greater collaboration between the United States, Spain, the United Kingdom, and Germany, with collaboration frequencies of 4 and 3, respectively (Figure 7). As for the number of documents published individually or through collaboration, the United States again led the ranking with a total of twenty-two articles, twenty authored individually, and two in collaboration; followed by Spain with seventeen articles, thirteen authored individually and four in collaboration; and the United Kingdom with fourteen, six published individually and eight in collaboration with other countries (Figure 8).

### 3.6. Most Productive Institutions

**Figure 9:**
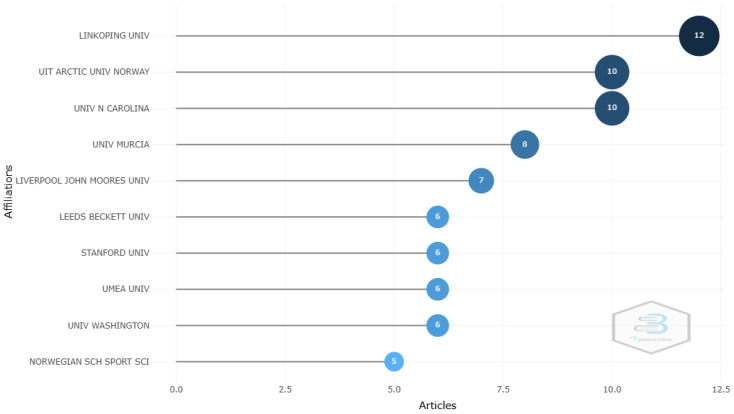
Most relevant affiliations.

Authors from 282 affiliations published research on women’s football. The five most relevant institutions, based on the number of published articles, were Linköping University (Sweden) (n = 12), The Arctic University of Norway (n = 10), The University of North Carolina (USA) (n = 10), The University of Murcia (Spain) (n = 8), and Liverpool John Moores University (UK) (n = 7). These institutions were from the five most productive countries (Figure 9).

### 3.7. Collaboration and Co-Citation Network Among Authors

**Figure 10:**
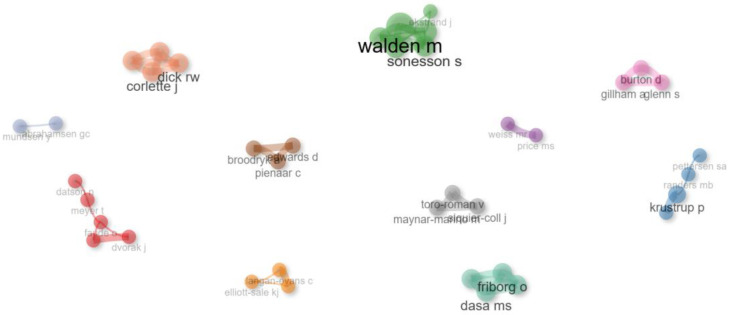
Collaboration visualization network among authors (co-authorships).

The analysis of the academic collaboration network among authors who had researched women’s football generated a visualization map to interpret collaborative dynamics [38]. The map is represented by different colors, each corresponding to a collaboration group. The dots or nodes represent each author who participated in the publications from the dataset being analyzed [39,40], and the size of the node indicates the influence of the author within the collaboration network. The lines represent the links between authors, and their thickness reflects the strength of the collaboration [41,42]. Figure 10 shows the 39 most prolific co-authors based on the number of publications, forming a network of 11 independent collaboration groups. The largest group included six authors, led by Walden and Akerlund, followed by a group of five authors with similar influence. Additionally, notable groups with four authors each were led by Krustrup; Dasa, Friborg, Kristoffersen, and Rosenvinge; and Corlette, Dick, Fuller, and Schmalz.

Regarding the co-citation network analysis, we examined how authors were cited together in other works, identifying relationships between articles, authors, or sources based on the number of times they were jointly cited in the same document [43].

**Figure 11:**
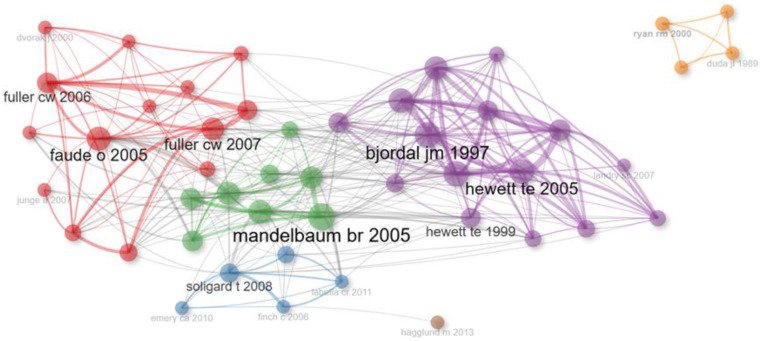
Author co-citation network in publications [22,44,45,46,47,48,49,50,51,52,53,54,55,56,57,58,59].

As shown in Figure 11, the largest set of connected elements in the women’s football co-citation visualization map consisted of seventeen authors in four different color groups. Metrics such as betweenness centrality (bet) and closeness centrality (cl) were considered, indicating the interrelation or influence on the researched topic. In each cluster, there was one author who acted as a bridge between the authors in their group and the rest. These were Faude (red cluster; bet = 40.18; cl = 0.011), Soligard (blue cluster; bet = 52.52; cl = 0.011), Arendt (purple cluster; bet = 73.78; cl = 0.011), and Mandelbaum (green cluster; bet = 170.26; cl = 0.011). According to the analysis data, Mandelbaum had the highest betweenness centrality, indicating that he may be one of the most influential authors, and his work is crucial for connecting the researched topic across different groups and the broader network.

### 3.8. Most Cited Articles

Continuing with the analysis of the most globally cited documents, we observed that the article titled “Prevention of non-contact anterior cruciate ligament injuries in soccer players. Part 1: Mechanisms of injury and underlying risk factors”, published in 2009 in the journal Knee Surgery, Sports Traumatology, Arthroscopy, had a total of 575 citations, averaging 35.94 citations per year.

Of the ten most cited articles, four focused exclusively on anterior cruciate ligament (ACL) injuries, their mechanisms, causes, and prevention, as these studies indicated that female soccer players were at a higher risk of suffering from this injury compared to men [16,17,21,60]. Two articles analyzed the incidence and risk factors for injuries in female soccer players to develop effective prevention strategies [22,61]. Similarly, another study compared the incidence, nature, and causes of injuries in soccer matches on natural grass versus new-generation artificial turf [44]. Another article reviewed the recovery strategies in soccer, particularly concerning the effects of active recovery and the anti-inflammatory response in elite female soccer players [62]. Finally, two articles analyzed the physical performance of female soccer players, examining the sprint characteristics and jumping abilities [63,64] and game-induced fatigue patterns in elite women’s soccer, focusing on pre- and post-match physical performance tests [18]. Table 6 presents the ten most cited articles, along with the total number of citations for each and the annual average.

### 3.9. Conceptual Structure Analysis

A conceptual structure analysis was developed to identify the main specific thematic trends within the topic of “women’s football” and its evolution over time as well as the factors driving their growth. A factor analysis was conducted using the author’s keywords (DE) and KeyWords Plus (ID). Both types of keywords provide precise information about the topics of the articles. On the one hand, DE are freely chosen words by the authors themselves or extracted from different thesauri, allowing them to accurately express the specific topics they considered most relevant in their research [65]. On the other hand, ID is automatically generated by an algorithm in the Web of Science (WoS) database in a more standardized way, based on the articles cited in each new publication. This automated process enables these keywords to provide search terms that go beyond the immediate topics addressed by the authors, offering a broader and more multidisciplinary view of the research field [66].

**Figure 12:**
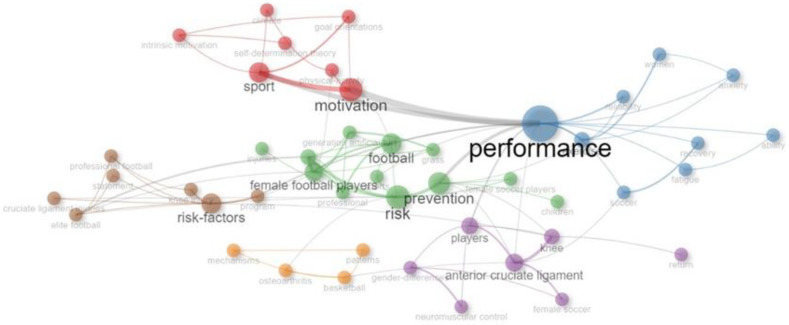
Co-occurrence network map of KeyWords Plus.

**Figure 13:**
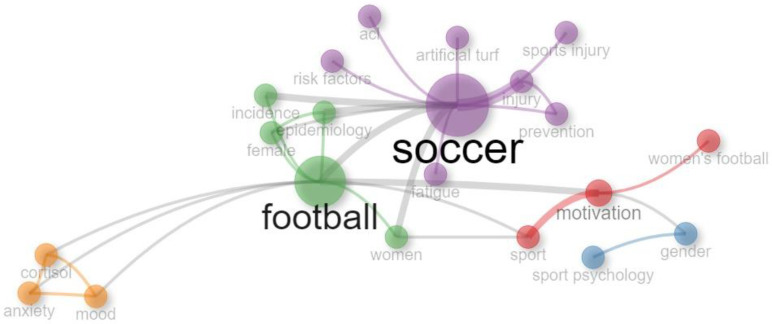
Co-occurrence network map of author’s keywords.

From the 127 documents analyzed, a total of 495 ID and 334 DE were obtained. A co-occurrence analysis was then performed, with a minimum occurrence value of 4 for both types of keywords. The network maps presented were created using the association strength method between different keywords and minimizing the number of links present in the map to select only the strongest connections between terms. This could be observed by the size of the nodes and the thickness of the linking lines (Figure 12 and Figure 13).

#### 3.9.1. KeyWords Plus

After the analysis, six clusters were identified within the 46 most frequently used keywords, with a minimum of 4 occurrences and a maximum of 24. The cluster with the most keywords (n = 12) was the green cluster, whose central terms were “risk”, “football”, “prevention”, and “female football players”, mainly addressing topics focused on injury prevention in football players. The other clusters presented topics related to motivation in sports or self-determination theory (red cluster), risk factors for knee and ACL injuries (brown and purple clusters), and sports performance (blue cluster). The ID “performance” had the highest number of occurrences (n = 24, blue cluster), followed by “motivation” and “sport” (n = 19; n = 15, red cluster) (Table 6). Analyzing the co-occurrence network of IDs (Figure 12), we observed words in each cluster with larger nodes, indicating that they appeared more frequently, tended to be more relevant in relation to others, and played a central role within the theme presented in each group.

#### 3.9.2. Author’s Keywords

Analyzing the DE, we observed that among the 20 most used keywords by authors, two terms stood out for their relevance and frequency of use: “soccer” and “football”, which were central to the research, with high occurrence values (n = 27; n = 22) as indicated by the size of their nodes (Table 6). The network map presented five clusters, two of which grouped a broader set of concepts (green cluster, n = 5; purple cluster, n = 7), representing greater thematic relevance, and therefore a research area with some dynamism and volume of investigation (Figure 13).

To conclude the analysis of the conceptual structure, the 15 most frequently used keywords were selected and ordered according to their occurrence value (Table 7). Based on the results, we observed that eight of the fifteen keywords were present in both types: “performance”, “motivation”, “sport”, “football”, “risk-factors”, “prevention”, “soccer”, and “epidemiology”. However, in most cases, the occurrence values were reversed—meaning that the ID (KeyWords Plus) with the highest values were chosen by the authors but were used less frequently, while DE (author’s keywords) were used more often but had lower occurrence values.

### 3.10. Three-Fields Plot Analysis

**Figure 14:**
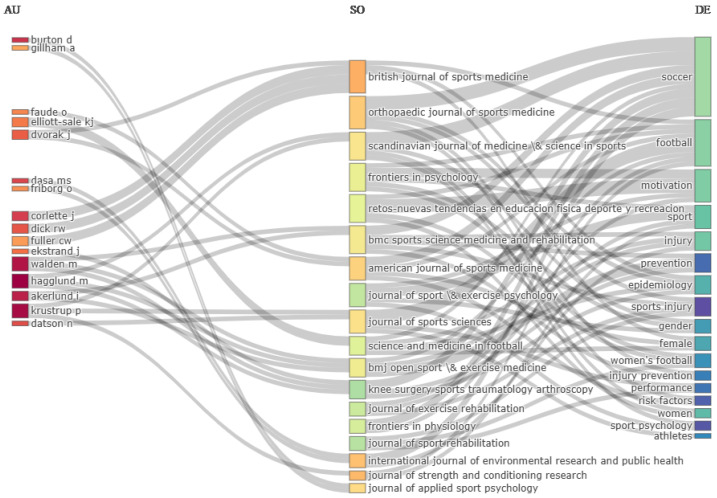
Three-fields plot: authors (AU), journals (SO), and author’s keywords (DE).

The bibliometric analysis using the three-fields plot revealed significant connections between the most prolific authors (AU), the most prominent journals (SO), and the author’s thematic descriptors (DE) in the field of research on women’s football (Figure 14).

Among the most influential authors, located on the left, Ekstrand, Hagglund, and Krustrup stood out, having published in high-impact journals such as the British Journal of Sports Medicine and the Scandinavian Journal of Medicine & Science in Sports, showing a clear preference for these outlets to disseminate their findings. These authors were particularly linked to research related to football, injury prevention, and physical performance in female athletes. On the other hand, authors like Gillham and Glenn published in the Orthopedic Journal of Sports Medicine, suggesting a more technical focus on orthopedic medicine in sports.

Regarding the DE (author keywords), located on the right side of the chart, the most researched topics in women’s football included terms such as “injury”, “risk factors”, “performance”, and specifically “women’s football”. These keywords were strongly connected to the mentioned journals, suggesting that most studies in this area focused on analyzing sports injuries and prevention strategies, with a significant emphasis on improving athletic performance in female players.

The most prominent journals in this field, positioned in the center of the figure, were the British Journal of Sports Medicine, Journal of Sports Sciences, and the American Journal of Sports Medicine. These journals acted as central nodes for the dissemination of research on women’s football, especially on topics related to epidemiology and the prevention of injuries, and gender differences in sports performance. Additionally, the descriptor “gender” was closely linked to research on women’s football, reinforcing the growing interest in gender issues in high-performance sports.

## 4. Discussion

This study aimed to analyze the evolution of scientific production on women’s football over the last 30 years, identifying the most frequent research topics and the main collaboration networks between the authors and institutions. Based on bibliometric data extracted from the Web of Science database, it was found that knowledge generated on this topic had grown considerably, especially in the last decade, coinciding with the increasing popularization and professionalization of women’s football globally, as suggested by high-profile events such as the FIFA Women’s World Cup (2019, 2023), the Olympic Games (2021, 2024), and the UEFA Women’s Champions League. The upward trend in article publication appears to align with institutional efforts to promote gender equality in sports, increasing the visibility of women in this field and simultaneously stimulating research in areas such as sports performance, motivation, psychosocial factors, injury incidence, and prevention [24,26].

### 4.1. Scientific Production and International Collaboration

The results show that women’s football has garnered growing academic interest since the 1990s, although the analysis revealed that until 2000, the number of articles was still low, with only three publications. The inclusion of women’s football in the 1996 Atlanta Olympic Games and the 1999 FIFA Women’s World Cup, held in the United States, may have laid the groundwork for subsequent academic interest, with sustained growth in scientific research observed [11]. By 2010, only 23 publications on the subject were recorded, but from 2011 onward, with the FIFA Women’s World Cup held in Germany, the infrastructure and organization reflected a shift toward the greater professionalization of women’s football, attracting many more live spectators and TV viewers. During that year and the following years, scientific production increased significantly, reaching 9 articles in 2013 and 12 articles in 2017, tripling the output of the previous decade, with a total of 52 articles. This growth aligns with the impact of key sporting events and increased institutional investment in women’s football and suggests that advancements in the sport’s professionalization may have directly influenced academic research, particularly on injury incidence [16,67,68], prevention [69,70,71], and recovery [72,73] as well as critical aspects for performance improvement, as reflected in studies by Jackman et al. [74], Krustrup et al. [18], Leyhr et al. [75], Shalfawi et al. [76], Tharawadeepimuk and Wongsawat [77], and Tounsi et al. [78], all focused on female football players.

The current decade (2020–2024) has been the most prolific in terms of scientific production, with a total of 55 publications to date, with 2022 being the year with the highest number of articles published, totaling 15. This increase coincides with the success of the 2019 FIFA Women’s World Cup in France, which broke audience records and solidified the global interest in women’s football. This event not only raised the sport’s visibility, but also sparked interest in other highly relevant topics, such as the rate of concussions in women’s football [79,80,81], to ensure player safety and understand the specific risks and effects on women. Other significant topics include the importance of psychological factors in performance, injury prevention, and recovery [20,82,83,84,85]; the levels of motivation and competitiveness [86,87]; gender differences in the physiology and biomechanics of female footballers [88,89,90,91,92,93]; and current issues such as misogyny in women’s football through social media [94]. Support from professional leagues and brands has also created a more robust research environment, reflected in the diversification of the areas studied in the past decade’s publications.

Research on women’s football has also been marked by strong international collaboration, as shown by the country collaboration map and co-authorship network analysis, reflecting the global nature of the sport and the interest it generates within the international scientific community. The United States, the United Kingdom, and Spain stand out as the countries with the highest scientific output, reflecting their respective progress in promoting women’s football at both the institutional and sporting levels. The fact that the United States leads the production of articles is consistent with its dominant position in women’s football at a competitive level, being four-time world champions, where investment in women’s leagues and institutional support, both academically and athletically, have been crucial to its development [95,96,97]. Moreover, institutions like the University of North Carolina and Stanford University have been leaders in research related to the health and performance of female footballers, reflecting the support of elite universities for the growth of knowledge in this field. Similarly, the contributions of European countries like Spain, the United Kingdom, Norway, Sweden, and Germany, among others, show recent growth in investment in national leagues and youth development programs [98,99,100,101]. Universities like Linköping in Sweden, the Arctic University of Norway, and the University of Murcia in Spain have been key institutions in the production of scientific research on women’s football, with a focus on injury prevention and sports physiology, establishing themselves as influential research centers in Europe. This phenomenon can also be explained by the expansion of international collaboration networks, facilitating the exchange of knowledge and best practices across regions.

### 4.2. Most Relevant Journal and Authors

The analysis of the most relevant journals in the scientific production on women’s football showed a notable concentration in a few publications, which is consistent with Bradford’s law, stating that a small number of journals tended to concentrate most of the published articles on a specific topic. In this case, the British Journal of Sports Medicine stood out as the journal with the highest number of articles, reflecting its relevance in the field of sports medicine and its influence on research into this sport. This journal is a key point for disseminating research on the incidence, characteristics, and risk factors for injury prevention [17,44,45,61,102,103,104], providing valuable data on the frequency and type of injuries in elite players, with a particular focus on knee injuries, especially anterior cruciate ligament (ACL) injuries, whose incidence is more than twice as high in women compared to men, remaining a priority in this group.

Other journals like the Journal of Strength and Conditioning Research and the International Journal of Environmental Research and Public Health have also made significant contributions to the literature on women’s football, though with a more specialized focus on physical conditioning, as revealed by studies from Dasa et al. [88], Krustrup et al. [18], Palmer et al. [105], Shalfawi et al. [76], and Van Den Tillaar [106], which analyzed physical performance by comparing different training methods and assess their effectiveness. Additionally, these journals explore psychological factors that influence sports performance, providing insights into their impact on players or establishing differences between the psychological profiles of professionals and amateurs [20,83,85]. This diversity in the approaches of the most influential journals highlights the multidisciplinary nature of research on women’s football.

Among the most prolific authors, Hägglund, Waldén, and Krustrup each stood out with three publications in this field, reflecting the significance of the topics these researchers focused on, particularly injury prevention and physical performance in women’s football—two critical research areas for athlete health and performance optimization. The fact that only 6% of authors had produced three articles indicates a high level of specialization in the field, with Krustrup being the most impactful author, boasting an H-index of 3 and a total of 176 citations. His notable contributions, especially in the realm of physical performance, game-induced fatigue, and the physiology of elite female football players [18,74,84], have provided valuable insights into the physical performance differences between men and women in football. These findings have helped justify the need for specific training strategies for female athletes. The high citation count reflects the importance of these findings and their utility for researchers, coaches, and sports professionals.

On the other hand, Hägglund and Waldén’s focus on ACL injury prevention, a recurring theme in the literature on women’s football, has been crucial in advancing knowledge about the causes of this injury and strategies to reduce its incidence [16,72,107]. These studies have had a significant impact not only in academia, but also in sports practice, influencing the design of training and prevention programs in women’s leagues worldwide.

### 4.3. Collaboration and Co-Citation Network Among Authors

The co-citation network highlights the thematic specialization in the field of women’s football, with key authors acting as central nodes in different research areas. This reflects the importance of figures such as Mandelbaum, Arendt, Soligard, and Faude, who have significantly contributed to the interconnection of studies on injury prevention and sports health [22,46,47,108]. The fact that these authors are frequently cited together in many articles suggests that their work has been fundamental in creating a cohesive body of knowledge on injury prevention, a crucial topic given the high risk of injuries in women’s football.

Thus, there is a well-established international collaboration structure, where certain authors have not only connected different research groups and contributed with high-impact publications but have also facilitated the creation of collaborative networks. These networks have expanded knowledge and applied their findings in global sports contexts, enriching the literature and providing a broader, multidisciplinary perspective on the challenges and opportunities faced by women’s football as it continues to grow.

### 4.4. Main Research Areas

The keyword analysis indicates that terms such as “performance”, “motivation”, “sport”, and “risk” are strongly associated with scientific production, and are thus linked to the most researched topics: injury prevention, physical performance, and sports motivation, which are directly tied to the professionalization process experienced by women’s football. The most cited studies focused on ACL injuries [17,21,60], one of the most pressing concerns, coinciding with the increasing number of players participating in high-level competitions.

The rise in research on sports motivation and the emotional well-being of female players since 2015 reflects the growing importance of women’s football as a platform for the empowerment of women in sports, coinciding with the boom in professional women’s leagues and increased institutional and commercial support for women’s sports [20,82,84,85,109]. These topics are not only important from a sports perspective, but also from a psychological and social viewpoint, as women’s football has historically been a way for women to challenge gender stereotypes and find motivation in developing their sports identity.

Despite the significant growth in research, there are still underrepresented areas, as indicated by previous studies [24,26]. Match analysis, specialized training for professional female players, and biomechanics applied to women’s football are areas that require more attention, especially considering the gender differences in terms of physiology and biomechanics. Additionally, while there have been significant advances in research on performance and injury prevention, the literature still lacks studies that have addressed deeper psychological aspects such as self-efficacy, leadership in female teams, and the impact of socioeconomic environments on the professional careers of female players.

## 5. Limitations

Finally, this study has some limitations that should be considered. First, it focused solely on the Web of Science database, which may have limited the inclusion of important studies published on other platforms such as Scopus or Google Scholar. In fact, there is a 2024 article that addresses the same topic using only the Scopus database, indicating a different, possibly complementary, approach in terms of the coverage of publications, journals, and relevant authors. Second, this database selection bias could have influenced the results, especially in terms of citations and the geographic scope of the studies included.

## 6. Conclusions

In summary, this bibliometric study has demonstrated significant growth in scientific production related to women’s soccer over the past three decades, identifying key research trends that span from physical performance and injury prevention to more recent topics such as sports motivation and gender equality in this field. The analysis revealed that the United States, the United Kingdom, and Spain lead research in this area, with notable international collaboration among institutions and authors from diverse regions. However, it is necessary to expand underexplored areas related to professional performance such as gender-specific biomechanical and physiological differences that can inform tailored training and recovery programs as well as the impact of concussions, given their high incidence in women. Moreover, it is crucial to examine the influence of psychological factors, such as emotional well-being and team cohesion on sports performance, along with the repercussions of harassment and misogyny on social media, which affect the sports environment and safety of women athletes. These areas of research are essential for advancing comprehensive support and the professionalization of women’s soccer. Therefore, this work lays the foundation for future research that broadens the scope of bibliometric analysis by incorporating other databases and addressing the gaps identified in the existing literature.

## Figures and Tables

**Table 1:** Search terms by keywords and database.

Keywords by Database
Web of Science
TS = ((“female soccer” OR “female soccer players” OR “women’s football” OR “girls football players” OR “female football” OR “female football players”) AND (“motivation” OR “cause” OR “motive”))

**Table 2:** Main categories and variables analyzed.

Measures	Items	Analysis
Overview	Main information	RStudio/Bibliometrix/Biblioshiny
	Scientific production per year	
	Average citations per year Three-field plot diagram	
Sources	Most relevant journals	RStudio/Bibliometrix/Biblioshiny
	Bradford’s law	
Authors	Most relevant authors Lotka’s law	RStudio/Bibliometrix/Biblioshiny
Countries	Corresponding author’s countries Scientific production by country Most cited countries	RStudio/Bibliometrix/Biblioshiny
Affiliations	Most relevant institutions	VOSviewer
Social Structure	Collaboration map between countries Author collaboration network Author co-citation network	RStudio/Bibliometrix/Biblioshiny
Documents	Global most cited articles Frequent word network author’s keywords Keyword Plus	RStudio/Bibliometrix/Biblioshiny

**Table 3:** Search terms by keywords and database.

Main Information	Description	Outcomes
Time Interval	Years of Publication	1992–2024
Sources	Source Distribution	75
Documents	Total Number of Documents	127
Document Types	Articles	108
	Book Chapters	1
	Early Access Articles	1
	Review Articles	14
	Publication Retractions	1
	Proceedings	2
Keywords	KeyWords Plus (ID)	496
	Author Keywords (DE)	335
Authors	Number of Authors	508
	Authors with Individual Authorship	7
Collaboration Between Authors	Collaboration Index	4.34
	Authors per Document	4.16
	Co-authors per Document	4.56
	International Co-authorships (%)	26.77
Citations	Average Citations per Doc per Year (mean, max., min.)	3.32/35.94/0
	Total Average Citations (mean, max., min.)	36.54/575/0

**Table 4:** Descriptive information of the top ten most relevant sources.

Journals	NP	TC	H-Index	PY_Start	JIF Average	JIF 2023	Q
British Journal of Sports Medicine	9	949	8	2006	7.67	11.8	Q1
Journal of Strength and Conditioning Research	6	281	5	2010	2.54	2.5	Q2
International Journal of Environmental Research and Public Health	5	32	3	2020	4.002	4.61 *	Q1 *
Orthopedic Journal of Sports Medicine	5	28	4	2017	3.31	2.4	Q2
American Journal of Sports Medicine	4	535	4	2005	4.59	4.2	Q1
Journal of Applied Sport Psychology	4	195	4	2002	1.67	2.7	Q3
Journal of Sports Sciences	4	75	3	2006	2.36	2.3	Q2
Scandinavian Journal of Medicine & Science in Sports	4	116	3	2004	3.33	3.5	Q1
BMC Sports Science Medicine and Rehabilitation	3	104	2	2017	2.05	2.1	Q1
BMJ Open Sport & Exercise Medicine	3	25	2	2019	4.45	3.9	Q1

JIF: journal impact factor; NP: publication number; Q: JIF quartile; PY_start: year of first publication; TC: total citations. * Latest JIF 2021.

**Table 5:** Production of the 10 most relevant countries with the highest number of citations.

Countries	Production	Citations
USA	106	992
United Kingdom	58	479
Spain	55	645
Norway	44	438
Sweden	40	641
Germany	26	254
Portugal	19	50
France	16	313
Canada	15	238
Poland	15	28

**Table 6:** Global most cited articles.

Authors	Title	Journal	TC	TCY
Alentorn-Geli et al. (2009), [21]	Prevention of non-contact anterior cruciate ligament injuries in soccer players. Part 1: Mechanisms of injury and underlying risk factors	Knee Surgery, Sports Traumatology, Arthroscopy	575	35.94
Yu and Garrett (2007), [17]	Mechanisms of non-contact ACL injuries	British Journal of Sports Medicine	316	17.56
Ahldén et al. (2012), [60]	The Swedish National Anterior Cruciate Ligament Register	The American Journal of Sports Medicine	287	22.08
Nédélec et al. (2013), [62]	Recovery in soccer	Sports Medicine	215	17.92
Haugen and Buchheit (2015), [63]	Sprint running performance monitoring: Methodological	Sports Medicine	214	23.78
Waldén et al. (2011), [16]	Anterior cruciate ligament injury in elite football: a prospective three-cohort study	Knee Surgery, Sports Traumatology, Arthroscopy	200	14.29
Faude et al. (2005), [22]	Injuries in female soccer players. A prospective study in the German National League	The American Journal of Sports Medicine	185	9.25
Emery et al. (2015), [61]	Neuromuscular training injury prevention strategies in youth sport: A systematic review and meta-analysis	British Journal of Sports Medicine	176	17.60
Fuller et al. (2007), [44]	Comparison of the incidence, nature and cause of injuries sustained on grass and new generation artificial turf by male and female football players. Part 1: Match injuries	British Journal of Sports Medicine	161	8.94
Krustrup et al. (2010), [18]	Game-induced fatigue patterns in elite female soccer	Journal of Strength and Conditioning Research	137	9.13

TC: total citations; TCY: total citations per year.

**Table 7:** Occurrence values of the top 15 KeyWords Plus (ID) and author’s keywords (DE).

Rank	KeyWords Plus	n	Author’s Keywords	n
1	Performance	24	Soccer	27
2	Motivation	19	Football	22
3	Sport	15	Motivation	10
4	Risk	13	Injury	7
5	Football	12	Sport	7
6	Risk-factors	11	Women	6
7	Prevention	10	Epidemiology	5
8	Exercise	9	Female	5
9	Female Football Players	9	Gender	5
10	Players	9	Women’s Football	5
11	Anterior Cruciate Ligament	8	Athletes	4
12	Knee	8	Injury prevention	4
13	Soccer	8	Performance	4
14	Epidemiology	7	Prevention	4
15	Female Soccer Players	7	Risk-factors	4

## Data Availability

The datasets generated during and analyzed during the current study are available from the main author or the corresponding author on reasonable request.
